# A Rare Case of Kostmann Syndrome Presenting Difficult Airway Challenges and Patient Preparedness for Anesthesiologists

**DOI:** 10.7759/cureus.26996

**Published:** 2022-07-18

**Authors:** Vamsi Krishna Uppalapati, Ashok Chattoraj, Deb Sanjay Nag, Himanshu Kumar, Sharad Kumar

**Affiliations:** 1 Anesthesiology, Tata Main Hospital, Jamshedpur, IND; 2 Surgery, Tata Main Hospital, Jamshedpur, IND; 3 Anaesthesiology, Tata Main Hospital, Jamshedpur, IND

**Keywords:** video laryngoscope, difficult iv access, difficult airway, anesthesia, severe congenital neutropenia (scn), kostmann syndrome

## Abstract

Severe congenital neutropenia (SCN), commonly known as the Kostmann syndrome, is a rare and complex set of disorders defined by a lack of neutrophil maturation in the bone marrow, leading to life-threatening complications. This case report discusses a young adult patient scheduled for elective laparoscopic cholecystectomy. The patient presented with skin lesions which are a common scenario of Kostmann syndrome, but along with that, our patient posed challenges of short neck, limited neck extension, and gynecomastia. These additional conditions dramatically increased the challenges for anesthesiologists to address the anticipated difficult airway. The anticipated difficult airway challenges were handled by following the protocols of difficult airway guidelines 2022.

## Introduction

Sir R Kostmann, in the year 1956, first described this rare autosomal recessive congenital syndrome [1,2,3]. The Kostmann syndrome incidence rate is three to 8.5 cases per million, and less than 100 cases are reported globally [4]. Kostmann syndrome is also known as severe congenital neutropenia (SCN), a rare and complex set of disorders defined by a lack of neutrophil maturation in the bone marrow, leading to life-threatening complications [5]. The syndrome is diagnosed in the early years of life with complaints of recurrent bacterial infections. Dermatological and upper respiratory tract infections are also reported in people with Kostman syndrome. Infections of the skin, respiratory tract, and deep tissues are seen in children with SCN caused by the deletion of the HAX1 gene from the first few months of life [6]. The condition is characterized by a decreased neutrophil count, which might be accompanied by thrombocytosis, eosinophilia, or monocytosis. At the promyelocyte stage, myelocyte formation is interrupted in persons with Kostmann syndrome. Cognitive decline, neurodegeneration, and epilepsy are common side effects in people with Kostmann syndrome [7]. In this case report, we present a patient with Kostmann Syndrome having challenges of difficult airway and other physical deformities. These deformities present enormous challenges for anesthesiologists to secure IV access [2] and emergency surgical airway [3]. The report further discusses anesthesia management for overcoming the perioperative challenges and preparedness for addressing anticipated difficult airway

## Case presentation

**Figure 1:**
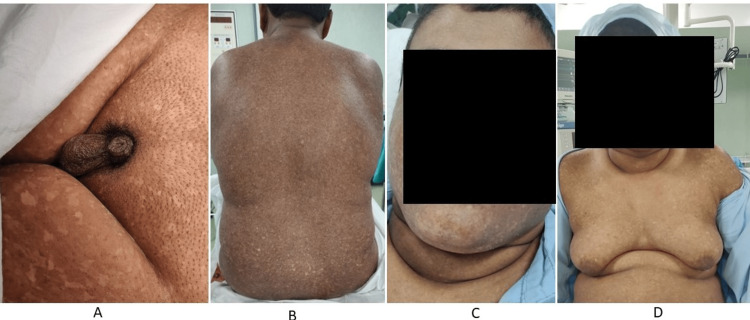
A: Microphallus, B: Skin lesions, C: Short neck with limited extension, D: Gynecomastia

A 25-year-old young adult with no past medical history was scheduled for elective laparoscopic cholecystectomy for symptomatic biliary colic. A thorough general physical examination and detailed study of current medical reports were conducted; his airway examination revealed adequate mouth opening with Mallampati grade 3; thyromental distance resulting less than three centimeters [8]. When the patient arrived for a pre-anesthetic checkup. The patient’s family history was positive for parental consanguinity [9,10], which unfortunately contributed heavily to being born with Kostmann syndrome. An extensive hematology workup showed significantly low neutrophil counts (15 gm% per cubic millimeter). The patient was a pre-diagnosed case of Kostmann syndrome and came for laparoscopic cholecystectomy. This was the first surgery for this patient with multiple physical deformities. The patient also went through severe emotional trauma that needed multimodal counseling sessions. Figure 1 below illustrates the physical and dermatological deformities.

 

## Discussion

Patients with Kostmann syndrome present multiple physical deformations; one of the serious challenges is skin lesions, making it difficult to secure IV access during anesthesia. Our patient presented with difficult IV access, so we placed a tourniquet on the desired extremity. But we could not make any vein prominent, so we switched to a continuous warm air technique using a blower. Vascular dilation in the limbs was caused by warm air [7]. We have all the tools and supplies necessary to perform the warm air procedure in our operating room. Two 18 gauze IV cannulas were securely secured as a result.

Neutrophil maturation is halted at the promyelocytic stage in patients with Kostmann syndrome [1,2]. From birth, children with severe neutropenia are more vulnerable to bacterial infections. When it comes to infections, our patient was no different from any other patient with Kostmann syndrome [1,2]. To avoid perioperative infections, broad-spectrum antibiotic injection ceftriaxone (1.5 gm) was provided when IV access was established.

Our patient also presented thyromental distance (TMD) of less than 6 cm, triple chin (receding mandible), short neck, and limited neck extension, making it an anticipated difficult airway scenario. We arranged the difficult airway cart consisting of various sized endotracheal tubes (ET), supraglottic airway devices, oral airways, various sized laryngoscope blades, and well-functioning suction apparatus.

**Figure 2:**
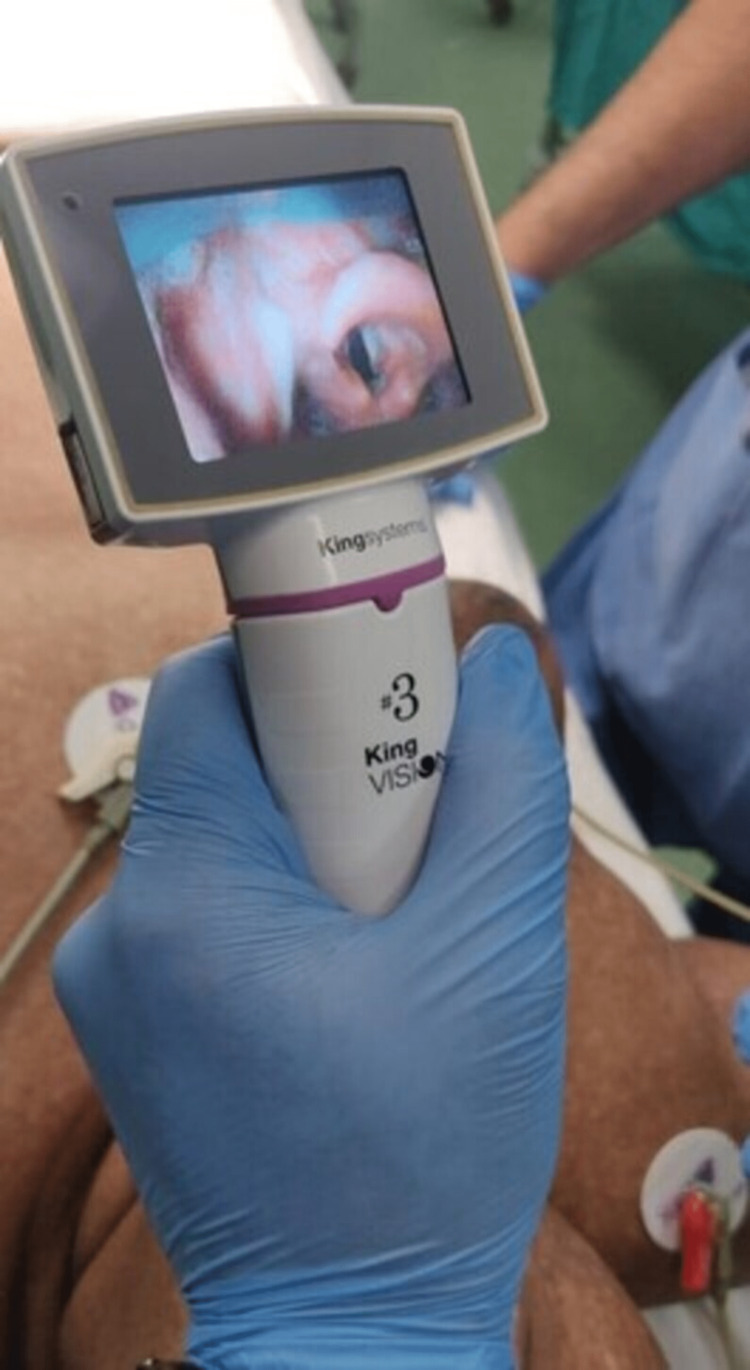
Video laryngoscope screen

Preoxygenation was done with 100% oxygen for three minutes. Induction was done with propofol (100 mg), our opioid of choice was fentanyl (100 mcg), and injection midazolam (1 mg). After confirming that we could ventilate the patient through a bag and mask, we paralyzed the patient with vecuronium (6 mg). Thorough ventilation for five minutes was carried out, and we attempted laryngoscopy using a video laryngoscope for better visualization. Figure 2 below illustrates the use of a video laryngoscope that also shows securing the ET tube. After confirming bilateral air entry through a five-point technique [11], we fixed the ET tube at 20 cm. Anesthesia was maintained with oxygen and nitrous (50-50).

Our inhalational agent of choice was sevoflurane (1.5 MAC to 1.71 Highest MAC). Injection of ondansetron (4 mg) was given as an antiemetic agent, and injection of dexamethasone (8 mg) to prevent postoperative nausea and vomiting. Standard American Society of Anesthesiologists (ASA) monitors [12] and temperature monitoring were used. Intraoperative hypothermia was prevented with warm IV fluids and warm air blowers. The surgery lasted for one hour, which was uneventful, and the patient was reversed with neostigmine (4500 mCg) and glycopyrrolate (650 mCg). After thorough suctioning, the patient was successfully extubated. Postoperative pain and further monitoring were as per the Aldrete score [13]. The patient was discharged on the second postoperative day without any complications. Postoperative investigations did not show any changes in neutrophil counts when compared to preoperative neutrophil counts.

## Conclusions

People born with syndromes are a result of genetic malformations. Some present more serious conditions than others as in the case of Kostmann syndrome, a genetic malformation that can also result from parental consanguinity like in our case. The common scenarios of Kostmann syndrome are skin lesions and significant neutropenia. In our case, the patient also presented other physical deformities such as a triple chin, short neck, limited neck extension, microphallus, and gynecomastia which could be conditions of another syndrome. However, these deformities present serious challenges for securing the difficult airway for anesthesiologists during surgery. The management of anesthesia by following the protocol mentioned in recent ASA-2022 difficult airway guidelines will reduce perioperative mortality and morbidity. 
